# Self-Organizing Surface-Initiated Polymerization of Multicomponent Photosystems: Stack Exchange with Fullerenes

**DOI:** 10.1002/open.201300004

**Published:** 2013-03-19

**Authors:** Altan Bolag, Hironobu Hayashi, Pierre Charbonnaz, Naomi Sakai, Stefan Matile

**Affiliations:** [a] Department of Organic Chemistry, University of Geneva30 Quai Ernest-Ansermet, 1211 Geneva (Switzerland) E-mail: stefan.matile@unige.ch Homepage: http://www.unige.ch/sciences/chiorg/matile/

**Keywords:** fullerenes, hydrazone exchange, oligothiophenes, photosystems, surface-initiated polymerization

The incorporation of spherical molecules into π-stack architectures of planar molecules is not a trivial task. The topological mismatch is significant. Ironically, one of the most attractive components in functional multicomponent architectures is a sphere. Namely, fullerenes, particularly C_60_, are of high interest to create electron-transporting pathways.^1^, ^2^ They are extensively used in the materials sciences, including most organic solar cells.^3^ Given this paramount importance, it is not surprising that much effort has been spent to accommodate the molecular spheres into uniform stacks of planar aromatics. The use of concave partners has been very productive.^4^ Numerous strategies have been conceived to attach the fullerene spheres covalently or noncovalently along linear architectures, including covalent polymers, π-stacks or coordination polymers.^5^ The alignment of fullerenes along oligothiophene stacks has received particular attention because of the importance of this architecture in organic solar cells.^6^ Highlights in this direction come from the groups of Bassani^7^ and Aida.^8^ Most difficult is the directional assembly of 1D fullerene channels. This is important to achieve directional electron transfer along redox gradients, as in biological photosystems. The leading example for this approach comes from the Imahori group.^9^ In the proposed strategy, fullerenes equipped with a ligand are coordinated to metalloporphyrin oligomers that have been grown directly on oxide surfaces.

Interested to learn how to grow functional multicomponent architectures directly on solid substrates, we have recently introduced self-organizing surface initiated polymerization (SOSIP) as a general synthetic method.^10^ In SOSIP, surface-initiated ring-opening disulfide-exchange polymerization is combined with molecular recognition to grow charge-transporting π-stacks with molecular-level precision on indium tin oxide surfaces (e.g., **1**; Figure 1, yellow boxes). To build multicomponent architectures, SOSIP has been expanded to include templated self-sorting (TSS)^11^, ^12^ and templated stack exchange (TSE).^13^, ^14^ For TSE, SOSIP architectures **1** are grown in the presence of templates on the surface (white box) and templates along the stack (grey circles, Figure 1). After SOSIP, the benzaldehyde templates are removed with hydroxylamine, and the large holes drilled into architectures **2** are filled by hydrazone formation with aldehydes of free choice.

**Figure 1:**
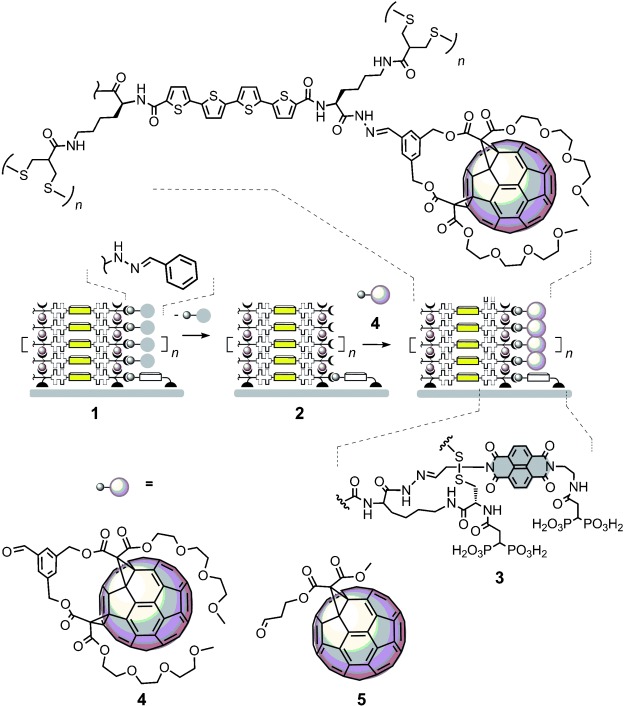
Schematic structure of oligothiophene-based SOSIP architectures before (**1**), during **(2**) and after templated stack exchange (**3**) with fullerene **4**.

SOSIP has been realized for several naphthalenediimide (NDI)^10^–^13^ and perylenediimide (PDI)^15^ stacks and, most recently, also oligothiophene stacks (i.e., **1**; Figure 1).^14^ Compatibility with TSE has been confirmed for triphenylamine, several core-substituted and core-expanded NDIs, PDIs, porphyrins and phthalocyanines.^13^, ^14^, ^16^ For the construction of multicomponent architectures with fullerenes, SOSIP was inconceivable at this point because the method is suited for stacks of planar aromatics and highly sensitive toward structural changes. TSE, in sharp contrast, is very tolerant to structural changes and is, in principle, compatible with the construction of strings of molecular spheres. Here we report facile access to oriented strings of fullerenes along oriented stacks of oligothiophenes, that is, double-channel photosystem **3**, by SOSIP-TSE with fullerene **4**.

Fullerene **4** was designed based on difficulties to achieve stack exchange with fullerene **5** (not shown). It contains an aromatic aldehyde to give stable hydrazones and two tri(ethylene glycol) (TEG) solubilizers to reach the concentrations in the polar aprotic solvents needed for TSE. The synthesis was based on protocols from the Nierengarten group. One TEG solubilizer **6** was attached to malonic acid **7** as described (Scheme 1).2c The resulting malonate monoester **8** was reacted with the primary alcohols in diol **9** to give bis-malonate **10**. Diol **9** was prepared as described by Nierengarten et al.2a Namely, 3,5-dibromobenzaldehyde **11** was protected as acetal **12** and formylated by treatment with first *tert*-butyl lithium (*t*BuLi) and then *N,N*-dimethylformamide (DMF). Reduction of the obtained dialdehyde **13** gave diol **9.** The regioselective double Bingel cyclopropanation2d of C_60_ with bis-malonates such as **10** has been developed early on in the Diederich group.2b The obtained macrocyclic bis-adduct **14** was deprotected to afford the final formyl-fullerene **4**.

**Scheme 1:**
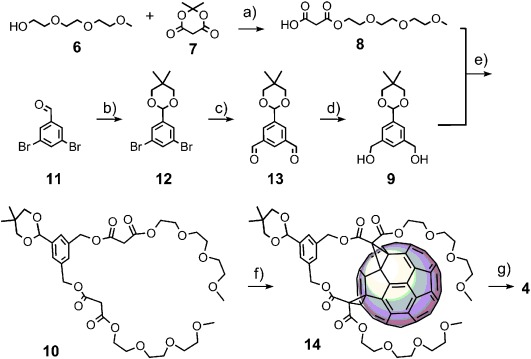
Synthesis of formyl-fullerene **4**. *Reagents and conditions*: a) 110 °C, 4 h, 80 %;2c b) 2,2-dimethyl-1,3-propanediol, C_6_H_6_, *p*TosOH cat., Δ, Dean–Stark trap, 97 %; c) *t*BuLi (4 equiv), THF, −78–0 °C, then DMF, −78–0 °C, then aq. 2 m HCl, 58 %; d) DIBAL-H, DCM, 0 °C, 97 %;2a e) DCC, DMAP, DCM, 0 °C to RT, 43 %; f) C_60_, DBU, I_2_, toluene, RT, 39 %; g) TFA, H_2_O, DCM, RT, 50 %.

Oligothiophene SOSIP was prepared, and the benzaldehyde template was removed as reported previously.^14^ Hydrazone bond formation between formyl-fullerene **4** and the photosystem **2** was evidenced spectroscopically by the appearance of characteristic fullerene absorption bands (Figure 2 A, Figure S7). Unlike the previously tested fullerene **5**, facile incorporation of fullerene **4** demonstrated the importance of its high solubility in polar organic solvent, and the tolerance of stack exchange to the structural variation. After apparent completion of the reaction in 2 h, the yield of stack exchange was estimated by comparing the absorbance of thiophene at 420 nm and fullerene at 320 nm before and after the stack exchange (see Supporting Information). The obtained 50 % yield is reasonable considering the three-dimensional bulk of the fullerenes. The fate of the remaining acyl hydrazines, if any, is unknown. Covalent addition to the fullerenes and oxidation during photocurrent generation are less likely given the mild conditions^17^ and the test-retest reliability of the photocurrent kinetics (see below).

**Figure 2:**
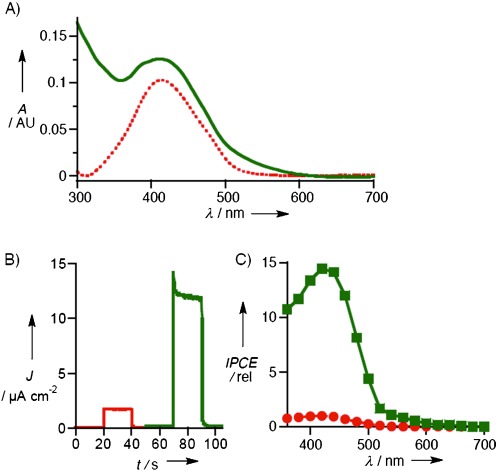
A) UV/Vis absorption spectra, B) photocurrent generation and C) action spectra of oligothiophene SOSIP photosystem **1** (—) and oligothiophene-fullerene photosystem **3** (—). Incident photon-to-current efficiency (IPCE) values are normalized against the IPCE of **1** at 420 nm (*A*=absorbance, AU=absorbance units, *J*=photocurrent density, rel=relative).

Photocurrent generation was examined under standard assay conditions. In brief, the photosystem was used as a working electrode together with a platinum wire counter electrode and a silver/silver chloride reference electrode. Triethanolamine (TEOA) was used as a mobile sacrificial hole acceptor; activities found with alternative hole acceptors such as the reversible carrier *p*-methoxyaniline di(2-ethylsulfonic acid) (MDESA) or ascorbic acid were analogous but overall clearly weaker.^15^ Irradiated with a solar simulator, oligothiophene–fullerene conjugate **3** generated much higher photocurrent than the photosystem **1** with only oligothiophenes (Figure 2 B). Repeated photocurrent generation gave unchanged kinetic profiles, suggesting that the decrease observed with **3** is not due to instability of the photosystem. Although conceivable with a HOMO energy around the −5.7 eV reported for the quaterthiophenes,^14^ irreversible oxidation of unreacted acyl hydrazines, if occurring, does therefore not account for the phenomenon. Repeatable photocurrent decrease with time thus originates most likely from biphasic saturation behavior somewhere along the charge-transporting pathways.

The action spectra (Figure 2 C) revealed about 15-times increased charge generation by the oligothiophene stack upon conjugation with fullerenes. Absorption and action spectra are reasonably well superimposable (Figure 2 A versus 2 C, green spectra). This observation suggested that not only the oligothiophenes but also the fullerenes contribute significantly to photocurrent generation. Decreasing rather than increasing activity with methyl viologen as electron acceptor in place of TEOA hole acceptors implied that the fullerenes are not just deposited on the surface of the SOSIP architecture but really form active stings along the oligothiophene stacks. This conclusion was further supported by the inability to deposit fullerenes in the absence of reactive hydrazides and aldehydes and the unproblematic detectability of fullerene reduction in the cyclic voltammogram of the film (similar to the ones measured in solution). These results are consistent with the formation of oligothiophene–fullerene heterojunctions.

In summary, herein we demonstrated the compatibility of the SOSIP-TSE strategy with fullerenes. Encouraged by these results, our current efforts focus on the preparation of various fullerene derivatives with different LUMO levels^1^ toward the construction of multicomponent heterojunction photosystems with built-in redox gradients.^13^, ^18^

## Supplementary material

As a service to our authors and readers, this journal provides supporting information supplied by the authors. Such materials are peer reviewed and may be re-organized for online delivery, but are not copy-edited or typeset. Technical support issues arising from supporting information (other than missing files) should be addressed to the authors.
