# Supplementary material for: Self-Organizing Surface-Initiated Polymerization of Multicomponent Photosystems: Stack Exchange with Fullerenes
Source: ChemistryOpen. 2013 Mar 19;2(2):55–7. doi: 10.1002/open.201300004 (PMC3646431; doi:10.1002/open.201300004)
Supplement: Supplementary file 1 [file open0002-0055-SD1.pdf]

ChemistryOPEN

Supporting Information

© 2013 The Authors. Published by Wiley-VCH Verlag GmbH & Co. KGaA, Weinheim

Self-Organizing Surface-Initiated Polymerization of Multicomponent Photosystems: Stack Exchange with Fullerenes

Altan Bolag, Hironobu Hayashi, Pierre Charbonnaz, Naomi Sakai, and Stefan Matile*[a]

open_201300004_sm_miscellaneous_information.pdf

Table of Content

1. Material and Methods S2
2. Synthesis S4
3. Stack Exchange S6
4. Photocurrent Measurements S8
5. Supporting Figures S9
6. Supporting References S15

1. Materials and Methods

As in [S1], Supporting Information. Briefly, reagents for synthesis were mostly purchased from Fluka, Sigma-Aldrich, and Across. Fullerene C_{60} was purchased from IoLiTec Ionic Liquids Technologies GmbH (Heilbronn, Germany). Indium tin-oxide (ITO) coated glass substrates were obtained from Präzisions Glas & Optik GmbH (Iserlohn, Germany).

Unless stated otherwise, column chromatography was carried out on silica gel 60 (Fluka, 40-63 μm). Analytical (TLC) and preparative thin layer chromatography (PTLC) was performed on silica gel 60 (Fluka, 0.2 mm) and silica gel GF (Analtech, 1 mm), respectively. UV-Vis spectra were recorded on a JASCO V-650 spectrophotometer equipped with a stirrer and a temperature controller (25 ± 0.1 ºC) and are reported as maximal absorption wavelength λ in nm (extinction coefficient ε in $M^{-1}cm^{-1}$). Melting points (Mp) were recorded on a heating table from Reichert (Austria) and uncorrected. IR spectra were recorded on a Perkin Elmer Spectrum One FT-IR spectrometer (ATR, Golden Gate) and are reported as wavenumbers v in cm^{-1} with band intensities indicated as s (strong), m (medium), w (weak), br (broad). ^{1}H and ^{13}C NMR spectra were recorded (as indicated) either on a Bruker 300 MHz, 400 MHz or 500 MHz spectrometer and are reported as chemical shifts (δ) in ppm relative to TMS (δ = 0). Spin multiplicities are reported as a singlet (s), doublet (d), triplet (t), quartet (q) and quintet (quint) with coupling constants (J) given in Hz, or multiplet (m). ^{1}H and ^{13}C resonances were assigned with the aid of additional information from 1D & 2D NMR spectra (H,H-COSY, DEPT 135, HSQC and HMBC). ESI-MS for the characterization of new compounds was performed on a Finnigan MAT SSQ 7000 instrument or an ESI API 150EX and are reported as mass-per-charge ratio m/z (intensity in %, [assignment]). ESI-HRMS for the characterization of new compounds were performed on a QSTAR Pulsar (AB/MDS Sciex) and are reported as mass-per-charge ratio m/z calculated and observed. Electrochemical measurements were done on an Electrochemical Analyzer with Picoamp booster and Faraday cage (CH Instruments 660C). Photocurrents were measured using a 150 W solar simulator (Newport) and an

Electrochemical Analyzer (CH Instruments 660C). The irradiation power was measured using a radiant power energy meter (Newport model 70260).

Abbreviations. Calcd: Calculated; DBU: 1,8-Diazabicyclo[5.4.0]undec-7-ene; DCC: *N,N'*-Dicyclohexylcarbodiimide; DCM: Dichloromethane; DMAP: 4-Dimethylaminopyridine; DTT: DL-Dithiothreitol; EtOAc: Ethyl acetate; ITO: Indium tin-oxide; rt: room temperature; TEOA: Triethanolamine; TFA: Trifluoroacetic acid.

2. Synthesis

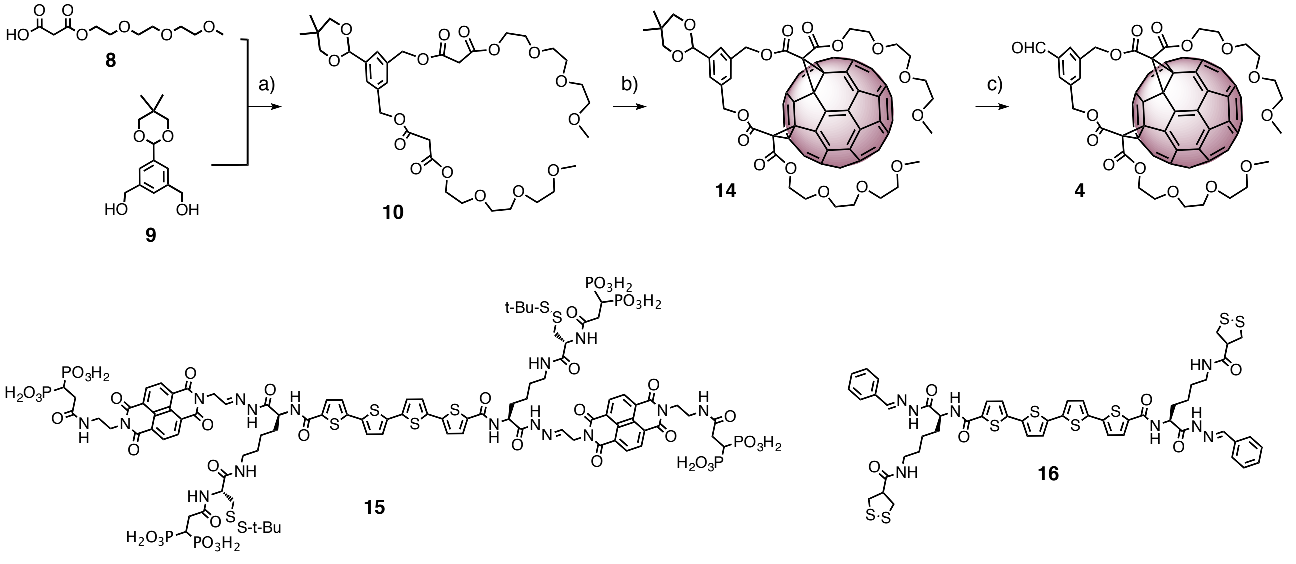

Scheme S1. a) DCC, DMAP, dry DCM, 18 h, 0 ºC to rt, 43%; b) C_{60}, DBU, I_2, toluene, 12 h, rt, 39%; c) TFA, H_2O, DCM, 4 h, rt, 50%.

Compounds 8 and **9** were synthesized according to procedures reported in [S2] and [S3], respectively.

Compound 10. To a solution of **9** (136 mg, 0.54 mmol), **8** (275 mg, 1.1 mmol) and DMAP (18 mg, 0.16 mmol) in dry DCM (15 ml) under an Ar atmosphere at 0 ºC was added a solution of DCC (220 mg, 1.07 mmol) in dry DCM. The mixture was allowed to reach to rt, stirred for 18 h, filtered and the solvent was evaporated. Diethyl ether was added to the residue and formed white solid was filtered and washed with diethyl ether. The filtrate was evaporated to dryness and silica gel column chromatography of the residue (DCM/EtOAc/MeOH 40:10:1) gave **10** (166 mg, 43%) as a colorless oil. R_f (DCM/EtOAc/MeOH 30:10:1): 0.22; IR (neat): 2950 (m), 2871 (m), 1731 (s), 1614 (w), 1456 (m), 1329 (m), 1269 (m), 1136 (s), 1102 (s), 1033 (s), 984 (s), 863 (m), 757 (w), 707 (w); ^{1}H NMR (300 MHz, $CDCl_3$): 7.52 (s, 2H), 7.39 (s, 1H), 5.45 (s, 1H), 5.24 (s, 4H), 4.34-4.30 (m, 4H), 3.82-3.54 (m, 24H), 3.52 (s, 4H), 3.42 (s, 6H), 1.34 (s, 3H), 0.86 (s, 3H); ^{13}C NMR (100 MHz,

$CDCl_3$): 166.6 (s), 166.4 (s), 139.7 (s), 136.0 (s), 128.7 (d), 126.4 (d), 101.2 (d), 77.9 (t), 72.1 (t), 70.8 (t), 69.0 (t), 67.0 (t), 64.8 (t), 59.3 (q), 41.6 (t), 30.5 (s), 23.3 (q), 22.1 (q); MS (ESI, MeOH/$CHCl_3$ 1:1): 739 (23, $[M+Na]^+$), 735 (100, $[M+NH_4]^+$); HRMS (ESI, +ve) Calcd for $C_{34}H_{56}O_{16}N$: 734.3594, found: 734.3621.

Compound 14. To a solution of C_{60} (169 mg, 0.23 mmol), **10** (166 mg, 0.23 mmol) and I_2 (119 mg, 0.47 mmol) in Toluene (300 ml) under Ar atmosphere, DBU (0.2 ml, 1.38 mmol) was added at rt. Resulting mixture was stirred for 12 h, then filtered through short plug of SiO_2 and washed with toluene and DCM. Silica gel column chromatography (DCM/MeOH 100:5) gave **14** (129 mg, 39%) as dark-red solid. R_f (DCM/MeOH 95:5): 0.58; Mp: 79-80 °C; IR: 2923 (s), 2857(s), 1749 (m), 1459 (m), 1377 (w), 1235 (m), 1107 (m), 1023 (w), 797 (w), 702 (w); ^{1}H NMR (400 MHz, $CDCl_3$): 7.50 (s, 1H), 7.45 (s, 2H), 5.90 (d, ^2J (H,H) = 13.1 Hz, 2H), 5.47 (s, 1H), 5.19 (d, ^2J (H,H) = 13.1 Hz, 2H), 4.56-4.45 (m, 4H), 3.83-3.53 (m, 24H), 3.37 (s, 6H), 1.31 (s, 3H), 0.84 (s, 3H); ^{13}C NMR (100 MHz, $CDCl_3$): 163.0 (s), 162.9 (s), 148.9 (s), 147.8 (s), 147.6 (s), 146.3 (s), 146.6 (s), 145.9 (s), 145.6 (s), 145.4 (s), 145.3 (s), 144.9 (s), 144.7 (s), 144.5 (s), 144.4 (s), 144.2 (s), 144.0 (s), 143.8 (s), 143.5 (s), 143.2 (s), 142.6 (s), 141.5 (s), 141.3 (s), 140.2 (s), 139.4 (s), 138.0 (s), 137.0 (s), 136.6 (s), 136.2 (s), 134.9 (s), 124.3 (d), 123.8 (d), 100.9 (d), 77.9 (t), 72.1 (t), 70.8 (t), 68.9 (t), 67.5 (t), 67.2 (t), 66.3 (t), 59.3 (q), 49.4 (s), 30.5 (s), 23.3 (q), 22.1 (q); MS (ESI, MeOH/$CHCl_3$ 1:1): 1451 (100, $[M+NH_4]^+$), 1435 (7, $[M+H]^+$), 1269 (18, $[M-C_7H_{15}O_4]^+$); HRMS (ESI, +ve) Calcd for $C_{94}H_{52}O_{16}N$: 1450.32806, found: 1450.32810.

Compound 4. A mixture of **14** (123 mg, 0.086 mmol), DCM (10 ml), TFA (4 ml) and H_2O (4 ml) was stirred at rt for 1.3 h. the organic layer was then washed with H_2O, dried (Na_2SO_4) and solvent evaporated. PTLC purification (DCM/MeOH 100:5) gave **4** (57 mg, 50%) as dark-red solid. R_f (DCM/MeOH 95:5): 0.51; Mp: 85-86 °C; IR (neat): 2874 (m), 1748 (s), 1701 (m), 1607 (w), 1439 (m), 1377 (m), 1418 (m), 1280 (m), 1234 (s), 1206 (s), 1142 (m), 1107 (m), 1064 (m), 1027 (m), 856 (w), 704 (w); ^{1}H NMR (300 MHz, $CDCl_3$): 10.1 (s, 2H), 7.84 (s, 2H), 7.81 (s, 1H), 5.94

(d, ^2J (H,H) = 13.3 Hz, 2H), 5.28 (d, ^2J (H,H) = 13.3 Hz, 2H), 4.58-4.48 (m, 4H), 3.81-3.53 (m, 20H), 3.36 (s, 6H); ^{13}C NMR (100 MHz, $CDCl_3$): 191.5 (d), 163.0 (s), 162.9 (s), 147.8 (s), 147.6 (s), 146.3 (s), 146.1 (s), 145.6 (s), 145.5 (s), 145.4 (s), 145.3 (s), 144.9 (s), 144.6 (s), 144.5 (s), 144.4 (s), 144.3 (s), 144.1 (s), 143.9 (s), 143.5 (s), 143.0 (s), 142.5 (s), 141.5 (s), 141.3 (s), 140.1 (s), 138.3 (s), 138.1 (s), 136.9 (s), 136.6 (s), 136.3 (s), 134.5 (s), 129.3 (d), 127.7 (d), 72.1 (t), 70.8 (t), 68.9 (t), 67.1 (t), 66.9 (t), 66.3 (t), 59.3 (q), 49.1 (s); MS (ESI, MeOH/$CHCl_3$ 1:1): 1370 (21, $[M+Na]^+$), 1365 (100, $[M+NH_4]^+$), 1347 (11, $[M+H]^+$), 1183 (67, $[M-C_7H_{15}O_4]^+$); HRMS (ESI, +ve) Calcd for $C_{89}H_{39}O_{15}$: 1347.22835, found: 1347.22840.

Compounds 15 and **16** were synthesized according to procedures reported in [S1].

3. Stack Exchange

Oligothiophene SOSIP photosystem **3** was prepared according to the procedures in [S1]. Briefly, cleaned ITO electrodes were coated with the initiator **15**, activated (DTT 20 mM in 10 mM aq. NH_4HCO_3, 1 h, rt) and shaken in a solution of propagator **16** (4-6 mM in $CHCl_3$/MeOH 3:1, with 0.1 M *i*-Pr_2NEt) for 24 h at rt. The obtained **1** was treated with aqueous NH_2OH (1 M) for 24 h at rt.

Thus prepared electrode **3** was dipped in a solution of fullerene **4** in DMSO and acetic acid (9 : 1, 12 mM) and shaken for 2 h at rt. The reaction was stopped when no more changes in UV-vis absorption spectra were observed.

The yield of stack exchange was roughly estimated from the molar ratio *R* obtained from the absorbances of the oligothiophene-fullerene photosystem **3** at 320 nm and 420 nm by using equations (S1-3):

$$A'_{\text{at 320 nm}} = \varepsilon'_{\text{oligothiophene}}[\text{oligothiophene}] + \varepsilon'_{\text{fullerene}}[\text{fullerene}] \qquad \text{(S1)}$$

$$A''_{\text{at 420 nm}} = \varepsilon''_{\text{oligothiophene}}[\text{oligothiophene}] + \varepsilon''_{\text{fullerene}}[\text{fullerene}] \qquad \text{(S2)}$$

$$R = [\text{fullerene}] / [\text{oligothiophene}] \quad \text{(S3)}$$

where A' and A'' are the absorbances at 320 nm and 420 nm, $\varepsilon'_{\text{oligothiophene}}$ and $\varepsilon''_{\text{oligothiophene}}$ are the molar absorption coefficients of oligothiophene at 320 nm and 420 nm, and $\varepsilon'_{\text{fullerene}}$ and $\varepsilon''_{\text{fullerene}}$ are the molar absorption coefficients of fullerene **4** at 320 nm and 420 nm, respectively. The following values were used oligothiophene: $\varepsilon' = 7 \times 10^3$ $M^{-1}cm^{-1}$ and $\varepsilon'' = 4 \times 10^4$ $M^{-1}cm^{-1}$, fullerene **4**: $\varepsilon' = 4.6 \times 10^4$ $M^{-1}cm^{-1}$ and $\varepsilon'' = 4 \times 10^3$ $M^{-1}cm^{-1}$. Quantitative yield $X = 100\%$ was assumed for a 2:1 ratio ($R = 2.0$). For the oligothiophene-fullerene photosystem **3**, $X \sim 50\%$ ($R = 1.0$) was obtained.

4. Photocurrent Measurement

Coated ITO electrodes were used as a working electrode with a Pt wire as a counter electrode and Ag/AgCl as a reference electrode. The electrodes were immersed in a deaerated (by bubbling Ar gas) aqueous solution of TEOA (50 mM) and Na_2SO_4 (0.1 M) and irradiated with a solar simulator (area of irradiation: $a \sim 1.0\ cm^2$). Changes in current upon on-off switching of irradiations were measured at 0 V vs Ag/AgCl unless stated. The power of irradiation was 42 $mWcm^{-2}$ unless stated otherwise.

Action Spectra. Photocurrent densities ($J_{sc} = I_{sc}/a$) were measured using TEOA (50 mM) and Na_2SO_4 (0.1 M) at 0 V vs Ag/AgCl upon excitation by monochromatic light (150 W Xe lamp with Oriel 1/8 m monochromator). The obtained current densities were converted into incident photon-to-current conversion efficiencies (*IPCE*) by using equation (S4).[S4]

$$IPCE = 1240 / \lambda \text{ (nm) x } J_{sc}/P_{in} \quad \text{(S4)}$$

5. Supporting Figures

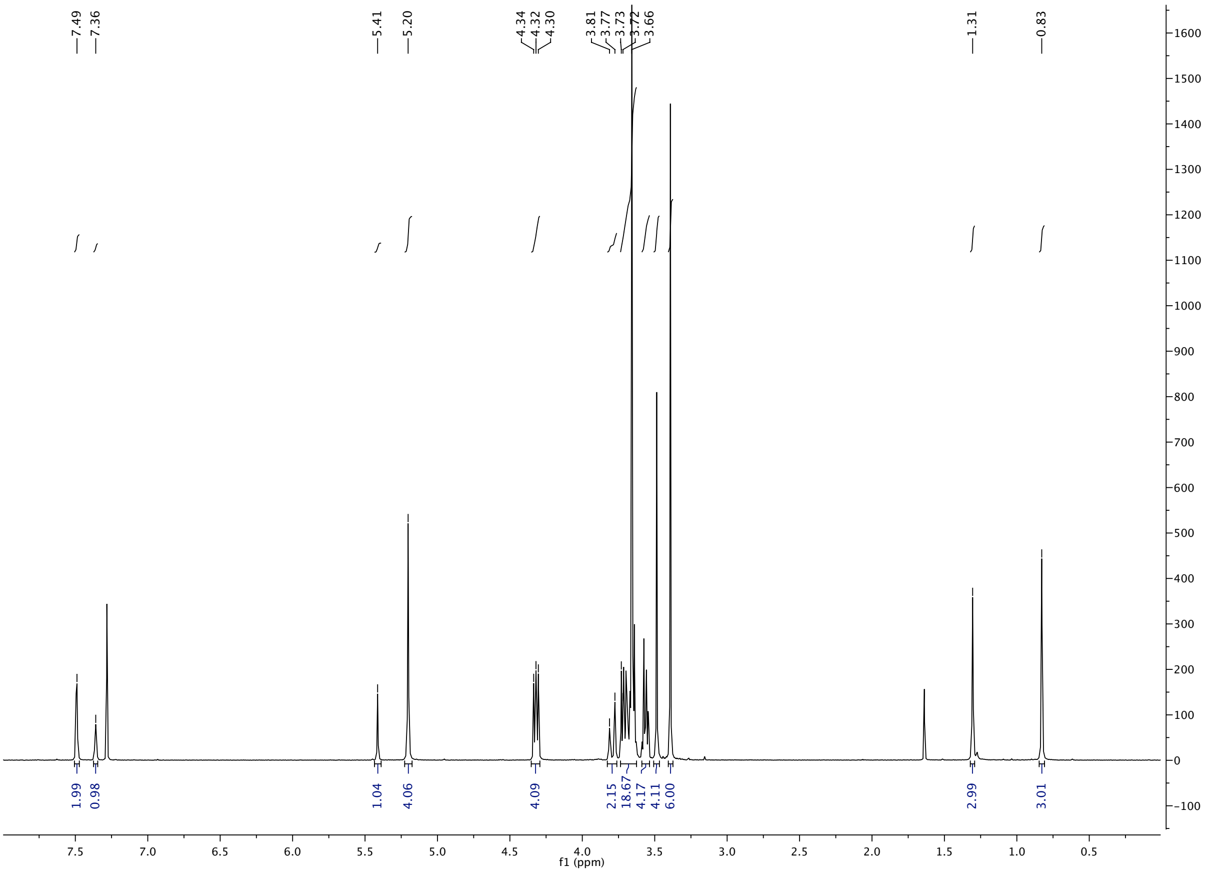

Figure S1. ^{1}H NMR spectrum of **10** in $CDCl_3$.

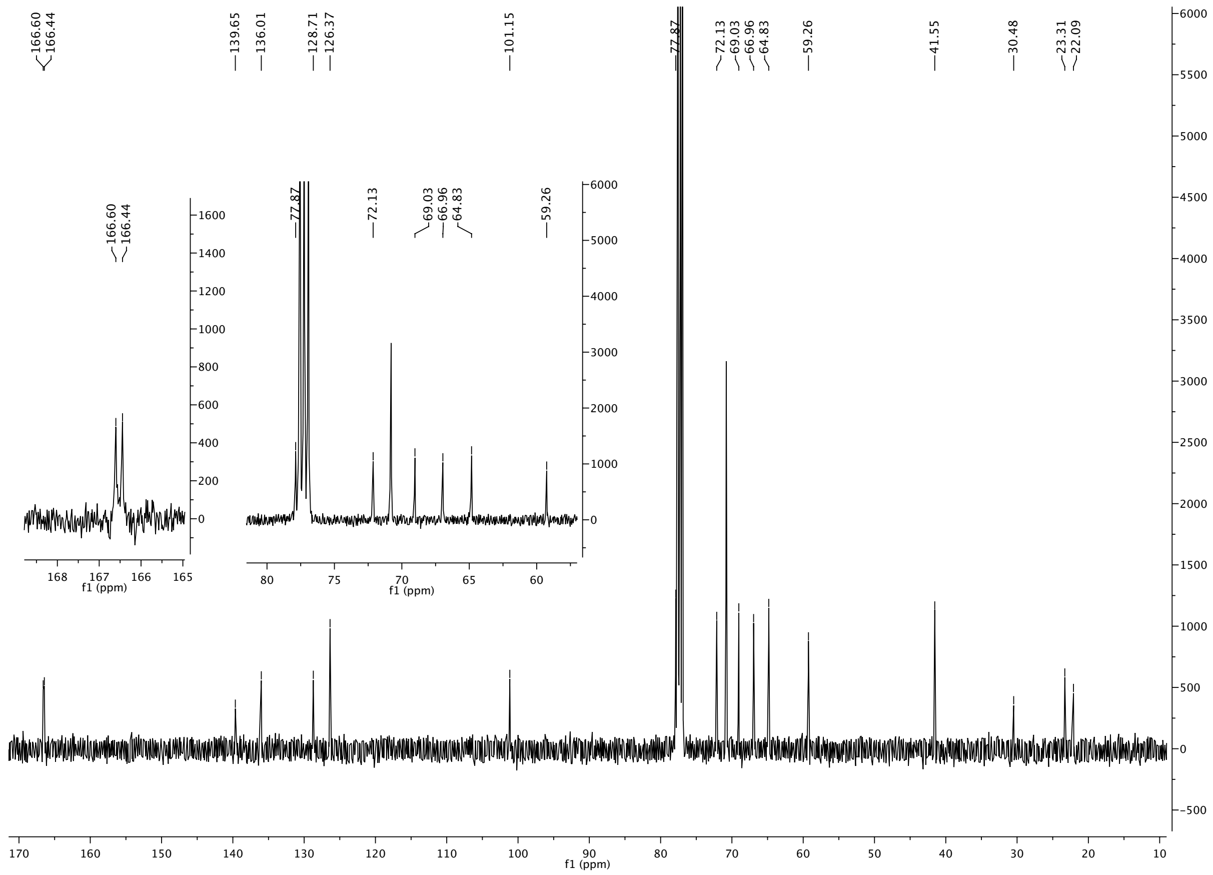

Figure S2. ^{13}C NMR spectrum of **10** in $CDCl_3$.

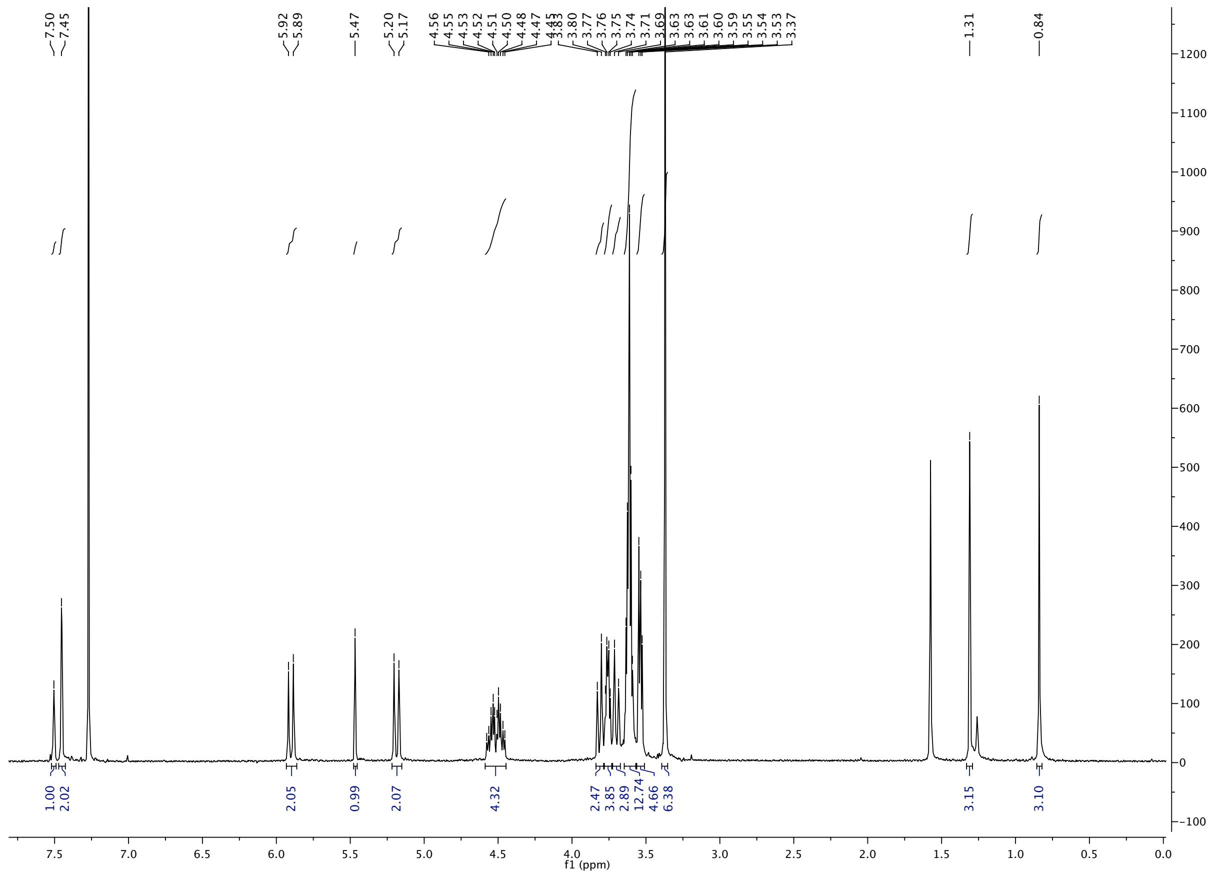

Figure S3. ^{1}H NMR spectrum of **14** in $CDCl_3$.

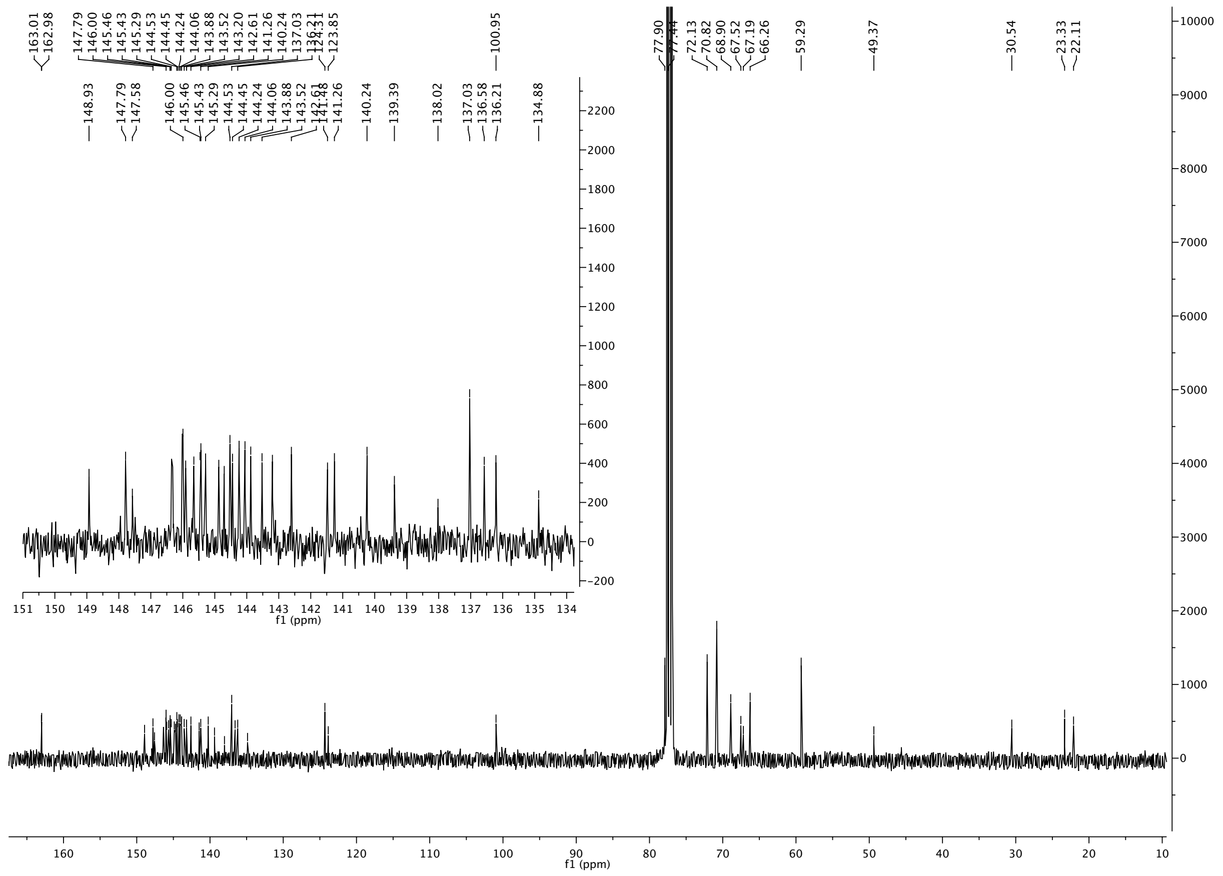

Figure S4. ^{13}C NMR spectrum of **14** in $CDCl_3$.

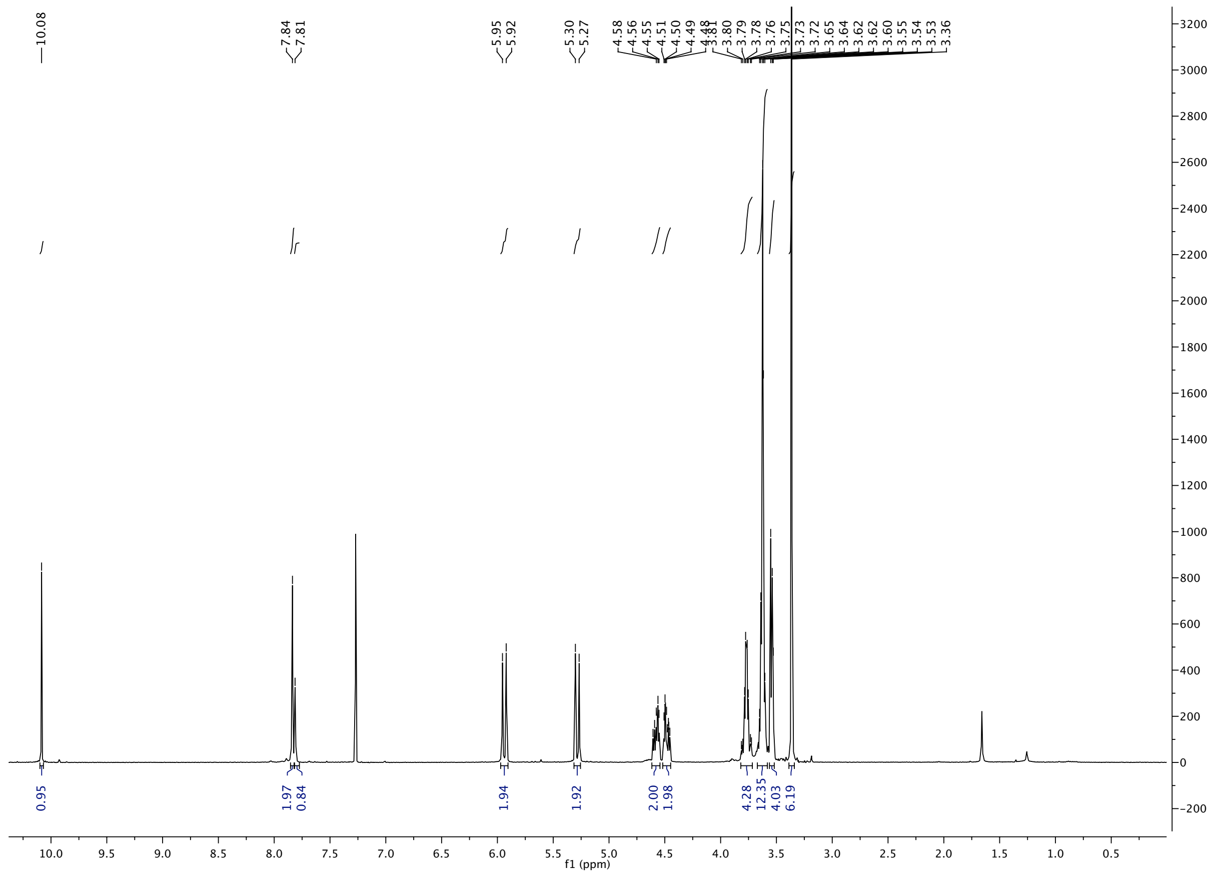

Figure S5. ^{1}H NMR spectrum of **4** in $CDCl_3$.

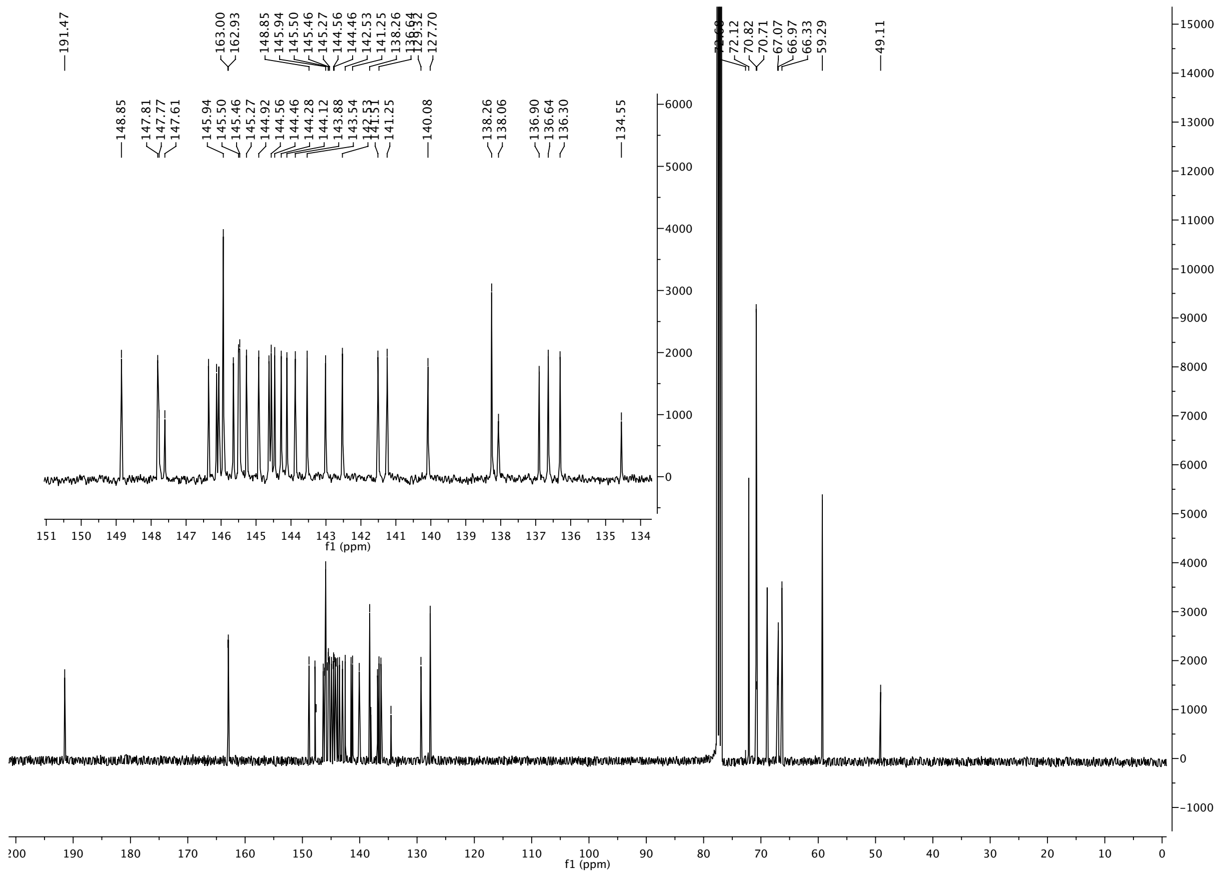

Figure S6. ^{13}C NMR spectrum of **4** in $CDCl_3$.

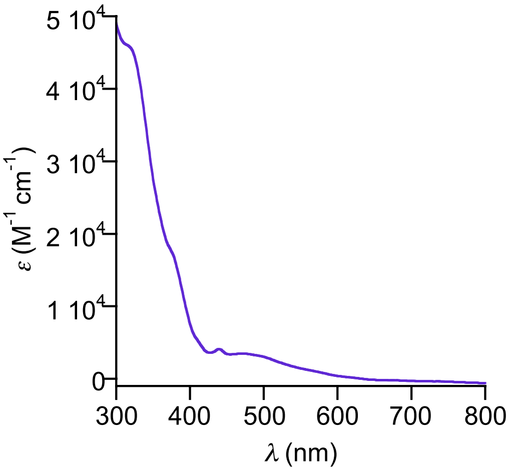

Figure S7. UV-Vis absorption spectrum of **4** in DMSO.

6. Supporting References

[S1] J. Areephong, E. Orentas, N. Sakai, S. Matile, *Chem. Commun.* **2012**, *48*, 10618–10620.

[S2] J.-P. Bourgeois, C. R. Woods, F. Cardullo, T. Habicher, J.-F. Nierengarten, R. Gehrig, F. Diederich, *Helv. Chim. Acta* **2001**, *84*, 1207–1226.

[S3] M. Urbani, J. Iehl, I. Osinska, R. Louis, M. Holler, J.-F. Nierengarten, *Eur. J. Org. Chem.* **2009**, *2009*, 3715–3725.

[S4] H. Yamada, H. Imahori, Y. Nishimura, I. Yamazaki, T. Ahn, S. Kim, D. Kim, S. Fukuzumi, *J. Am. Chem. Soc*. **2003**, *125*, 9129-9139.
